# Branch Retinal Vein Occlusion: Treatment Outcomes According to the Retinal Nonperfusion Area, Clinical Subtype, and Crossing Pattern

**DOI:** 10.1038/s41598-019-42982-5

**Published:** 2019-04-25

**Authors:** Yuko Iida-Miwa, Yuki Muraoka, Yuto Iida, Sotaro Ooto, Tomoaki Murakami, Kiyoshi Suzuma, Akitaka Tsujikawa

**Affiliations:** 1 Department of Ophthalmology and Visual Sciences, Kyoto University Graduate School of Medicine, 54 Shogoin Kawahara-cho, Sakyo-ku, Kyoto, Japan; 2 Department of Ophthalmology, Kagawa University Faculity of Medicine, Kagawa, Japan

**Keywords:** Retinal diseases, Outcomes research

## Abstract

This prospective study examined 58 eyes with branch retinal vein occlusion (BRVO) to investigate the effects of the nonperfusion area (NPA), clinical subtype, and crossing pattern on the 2-year outcomes of ranibizumab therapy for the macular edema (ME). All eyes received three initial monthly injections, followed by additional pro re nata (PRN) injections. The final best corrected visual acuity (BCVA) and ranibizumab injection number were not associated with the macular NPA or total NPA at baseline or month 12, and they showed no significant differences between the clinical subtypes. However, the incidence of neovascular changes was higher in the major BRVO group than in the macular BRVO group (*P* = 0.030). Twelve and 19 of the 34 eyes with major BRVO exhibited arterial overcrossing and venous overcrossing, respectively. At baseline, the total NPA did not differ according to the crossing pattern, however, the total NPA was significantly larger in the venous overcrossing group at month 12 (*P* = 0.047). At month 24, the incidence of neovascular changes was higher in the venous overcrossing group (*P* = 0.030). Following ranibizumab therapy for BRVO-associated ME, the clinical subtype and the arteriovenous crossing pattern may be associated with neovascular changes.

## Introduction

Branch retinal vein occlusion (BRVO) is the second-most frequent retinal vascular disorder^1–4^. In the majority of cases, BRVO occurs when a first- or second-order retinal vein focally occludes at an arteriovenous (AV) crossing^5–9^, and results in the development of macular edema (ME)^4,10^ and a retinal nonperfusion area (NPA)^4,10–12^ under upregulation of vascular endothelial growth factor (VEGF)^13,14^. NPA extending to the macular area can directly deteriorate the central vision^15–17^, while a large peripheral NPA can cause vitreous hemorrhage due to retinal or disc neovascularization in the chronic phase^3,4,10^.

Using ultra-widefield angiography, Prasad *et al*. reported that untreated NPA may be the source of the increase in VEGF that promotes ME^18^. In contrast, the RELATE trial^19^ and WAVE study^20^ reported that the addition of laser photocoagulation for NPA did not substantially affect the treatment burden or morphological outcomes of intravitreal ranibizumab injections for eyes with retinal vein occlusion. Therefore, the effects of macular and peripheral NPAs on the functional and morphological outcomes of anti-VEGF therapy for BRVO-associated ME may remain unclear.

Recent advances in optical coherence tomography (OCT) and OCT angiography have enabled detailed longitudinal and quantitative observations of retinal vasculature changes^16,21–23^. Investigations based on sequential thin sectioning with OCT^24^, and OCT angiography^25^ reported that the mechanism underlying BRVO and affecting the NPA size may differ according to the relative anatomical position of the crossing vessels. However, the duration from onset to OCT angiography examination and the duration of observation varied among patients in those studies because of the retrospective study designs; furthermore, the researchers were unable to include patients with macular BRVO or examine any neovascular changes^24,25^.

In the present prospective study, we included patients with both major BRVO and macular BRVO and obtained quantitative measurements of central macular NPAs using OCT angiography and total NPAs using ultra-widefield angiography. In addition, we investigated the effects of these NPA measurements, the clinical subtype of BRVO, and the vascular crossing pattern on the 2-year outcomes of ranibizumab treatment for ME, including the incidence of neovascular complications.

## Results

We included 58 eyes of 58 patients with treatment-naïve BRVO in the present study. Table 1 shows the clinical characteristics of the patient groups. At baseline, the mean symptom duration was 2.2 ± 1.4 months (range: 0–4 months). In accordance with previous reports^9,26^, we defined major BRVO as an occlusion of the temporal arcade vein or the branch that extended to the retinal periphery beyond the retinal vascular arcades^9^. We defined macular BRVO as an occlusion limited to a smaller venous tributary draining a section of the macula, located between the superior and inferior temporal arcades^26^. We included 34 eyes (58.6%) in a major BRVO group (the affected retinal areas are beyond the retinal vascular arcades) and 24 eyes (41.4%) in a macular BRVO group (the affected retinal areas are restricted within the retinal vascular arcades). Using OCT angiography images of the affected AV crossing, we included 32, 23, and three eyes in arterial overcrossing, venous overcrossing, and undetermined groups, respectively (Table 1). Intraclass correlation coefficients (ICCs) for the central macular NPA measurements in the superficial and deep plexuses were 0.992 and 0.998, respectively.

**Table 1:** Characteristics of Included Patients with Branch Retinal Vein Occlusion.

Number of patients (women/men)	38/20
Age (years)	69.1 ± 10.7
Number of patients with systemic hypertension (n, %)	24 (41.4%)
Number of patients with hyperlipidemia (n, %)	13 (22.4%)
Duration of symptoms (months)	2.2 ± 1.4
BRVO subtype (major BRVO/macular BRVO; n)	34/24
Arteriovenous crossing type at affected site (arterial overcrossing/venous overcrossing/undetermined; n)	32/23/3

Data are mean ± standard deviation unless otherwise indicated; BRVO, branch retinal vein occlusion.

### Time-Dependent Changes in Clinical Parameters

**Figure 1:**
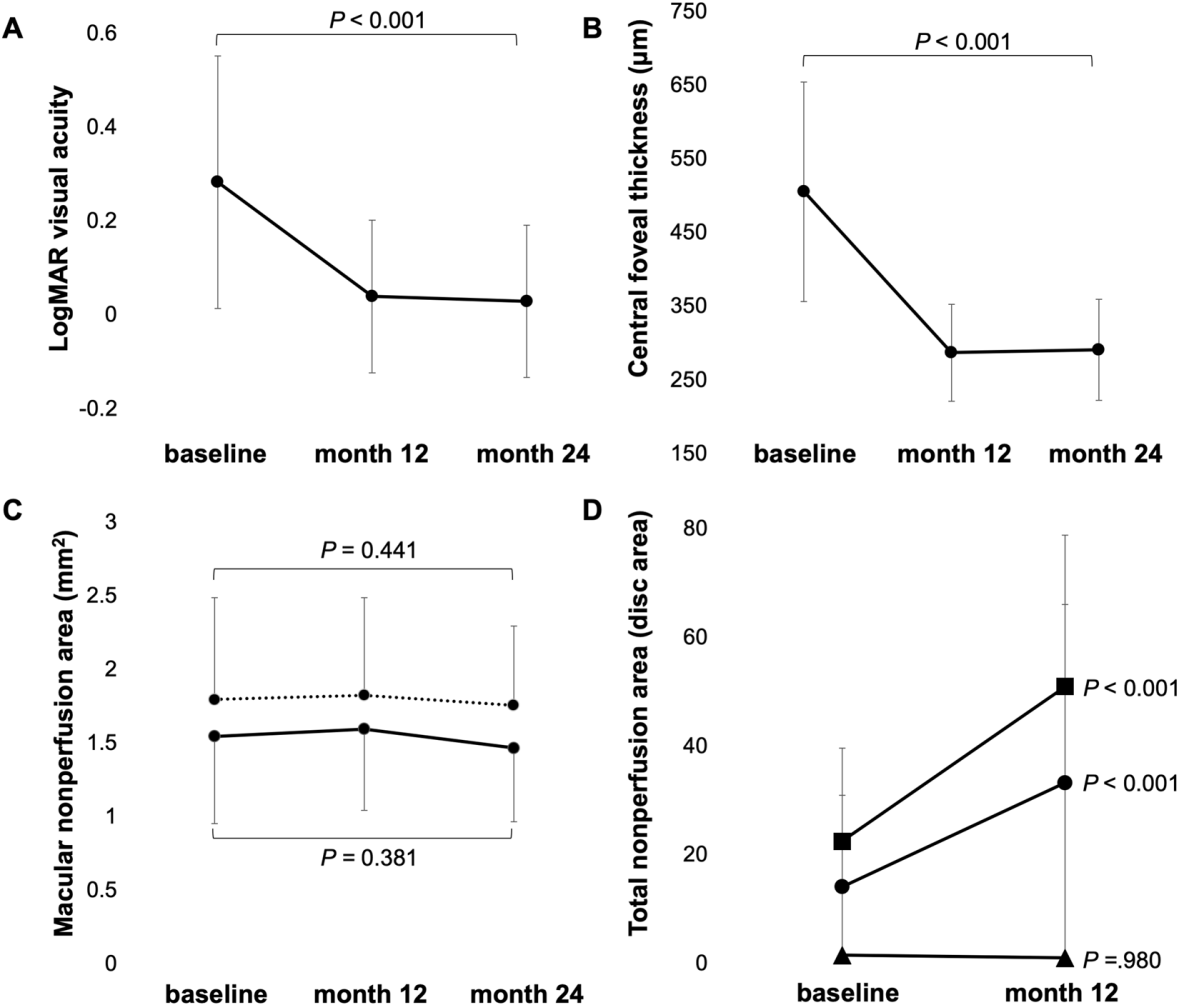
Time-dependent changes in the best corrected visual acuity (BCVA; **A**), central foveal thickness (CFT; **B**), macular nonperfusion area (NPA; **C**), and total NPA (**D**) in patients treated with ranibizumab injections for macular edema caused by branch retinal vein occlusion (BRVO). At month 24, BCVA (logarithm of the minimum angle of resolution units) has improved while CFT has significantly decreased (*P* < 0.001 vs. baseline measurements for both). In contrast, the macular NPA in the superficial capillary plexus (dotted line) and deep capillary plexus (solid line) has not changed (**C**). The total NPA has significantly increased in the overall patient sample (circle) and in the subgroup of patients with major BRVO (square), whereas it has not changed for patients with macular BRVO (triangle; **D**).

**Figure 2:**
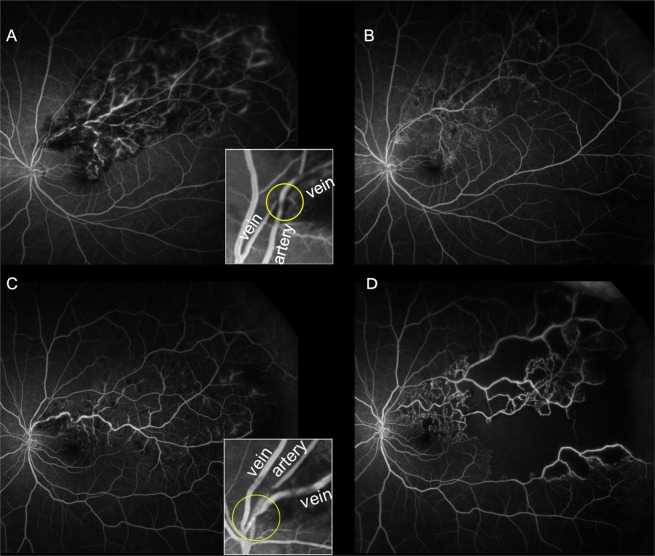
Enlargement of retinal nonperfusion areas (NPAs) according to the anatomical position of the retinal vessels at affected arteriovenous crossings in eyes with branch retinal vein occlusion (BRVO). Ultrawide-field fluorescein angiography showing an arterial overcrossing pattern (**A**,**B**) and a venous overcrossing pattern (**C**,**D**). (**A**,**C**) Images at baseline. (**B**,**D**) Images at month 12. Images inset in panels A, and C show enlargements of the affected crossing sites. In images for a representative case of venous overcrossing (**C**,**D**), a marked increase in NPA, especially in the periphery, can be observed. In contrast, NPA enlargement is unremarkable in representative images for a case of arterial overcrossing (**A**,**B**).

Table 2 and Fig. 1 show the time-dependent changes in best corrected visual acuity (BCVA), central foveal thickness (CFT), the central macular NPA, and the total NPA after the initial ranibizumab injections. At baseline, the mean BCVA (logarithm of the minimal angle of resolution [logMAR]) was 0.28 ± 0.27 and the mean CFT was 495.6 ± 141.0 µm. Relative to the baseline values, BCVA improved at both 12 and 24 months, while CFT significantly decreased at both time points (*P* < 0.001 for all). The central macular NPAs showed no significant changes from baseline to 12 or 24 months, while the total NPA was significantly larger at month 12 than at baseline in all eyes (*P* < 0.001, Table 2). Ultrawide-field fluorescein angiography performed at month 12 occasionally showed NPA enlargement, particularly in the peripheral retina of eyes with major BRVO (Fig. 2C,D). ICC for the total NPA measurements was 0.932.

**Table 2:** Changes at 12 Months and 24 Months after Initial Ranibizumab Injection.

	BL	12 M	24 M	*P* values		
				ANOVA	Post hoc	
					BLvs12M	BLvs24M
Mean logMAR VA	0.28 ± 0.27	0.03 ± 0.14	0.03 ± 0.16	<0.001	<0.001	<0.001
Foveal thickness (μm)	495.6 ± 141.0	286.1 ± 65.8	289.9 ± 68.4	<0.001	<0.001	<0.001
Total NPA (disc area)	14.0 ± 16.8	33.1 ± 32.9	—	N. A.	<0.001	N. A.
Central Macular NPA in SCP (mm^2^)	1.53 ± 0.59	1.59 ± 0.55	1.46 ± 0.50	0.891	N. A.	N. A.
Central Macular NPA in DCP (mm^2^)	1.79 ± 0.69	1.82 ± 0.66	1.75 ± 0.54	0.780	N. A.	N. A.

BL = baseline; VA = visual acuity; M = months; NPA = retinal nonperfusion area; ANOVA = repeated measures analysis of variance. To avoid segmentation errors, the baseline measurements of optical coherence tomography angiography were obtained after the initial resolution of macular edema.

### Prognostic Factors Associated with the Final Visual Acuity and Ranibizumab Injection Number

Table 3 shows the associations between the final BCVA and clinical factors at baseline and month12. The final BCVA (logMAR) was positively associated with age (*P* < 0.001), the BCVA at baseline and month 12 (*P* < 0.001 for both), the baseline CFT (*P* = 0.014), whereas it showed no association with the central macular NPA or total NPA at baseline or month 12.

**Table 3:** Associations Between Final Visual Acuity and Clinical Factors at Baseline and 12 Months after Initial Treatment.

	R	*P* value
Baseline		
Age	0.566	<0.001
LogMAR visual acuity	0.639	<0.001
Foveal thickness	0.323	0.014
Total NPA	0.073	0.596
Central macular NPA in the SCP	0.199	0.376
Central macular NPA in the DCP	0.090	0.692
12 months after initial treatment		
LogMAR visual acuity	0.738	<0.001
Foveal thickness	0.086	0.520
Total NPA	0.036	0.815
Central macular NPA in the SCP	0.306	0.055
Central macular NPA in the DCP	0.152	0.350
Total number of IVR injections at 12 months	0.009	0.944

IVR = intravitreal ranibizumab; LogMAR = logarithm of the minimum angle of resolution; NPA = retinal nonperfusion area. SCP = superficial capillary plexus; DCP = deep capillary plexus.

Table 4 shows the associations between the number of ranibizumab injections required and clinical factors at baseline and month12. The mean number of ranibizumab injections was 5.4 ± 2.2. No factor at baseline or month 12 was found to be associated with the number of ranibizumab injections.

**Table 4:** Associations Between Total Number of Intravitreal Ranibizumab Injections and Clinical Factors at Baseline and 12 Months after Initial Treatment.

	R	*P* value
Baseline		
Age	0.101	0.450
LogMAR visual acuity	0.231	0.083
Foveal thickness	0.164	0.223
Total NPA	0.006	0.965
Central macular NPA in SCP	0.133	0.555
Central macular NPA in DCP	0.120	0.595
12 months after initial treatment		
LogMAR visual acuity	0.037	0.781
Total NPA	0.061	0.689
Central macular NPA in SCP	0.044	0.787
Central macular NPA in DCP	0.060	0.713

LogMAR = logarithm of the minimum angle of resolution; NPA = retinal nonperfusion area; SCP = superficial capillary plexus; DCP = deep capillary plexus.

### Comparison of Outcomes Between the Major and Macular BRVO Groups

To confirm the insignificant associations between the total NPA and other clinical outcomes, we compared the clinical outcomes between the major BRVO and macular BRVO groups (Table 5).

**Table 5:** Comparison of Clinical Characteristics between Major Branch Retinal Vein Occlusion and Macular Branch Retinal Vein Occlusion.

	Major BRVO (34 eyes)	Macular BRVO (24 eyes)	*P* value
Baseline measurements			
LogMAR visual acuity	0.31 ± 0.320	0.24 ± 0.173	0.269
Foveal thickness (μm)	528.0 ± 147.3	450.5 ± 120.8	0.023
Total NPA (disc area)	22.3 ± 17.2	1.42 ± 2.36	<0.001
Central macular NPA in SCP (mm^2^)	1.64 ± 0.56	1.35 ± 0.62	0.271
Central macular NPA in DCP (mm^2^)	1.95 ± 0.68	1.51 ± 0.64	0.152
12 months after initial treatment			
LogMAR visual acuity	0.03 ± 0.18	0.04 ± 0.13	0.812
Total NPA (disc area)	50.8 ± 27.9	0.92 ± 2.11	<0.001
Central macular NPA in SCP (mm^2^)	1.65 ± 0.58	1.51 ± 0.53	0.445
Central Macular NPA in DCP (mm^2^)	1.92 ± 0.70	1.70 ± 0.60	0.295
24 months after initial treatment			
LogMAR visual acuity	0.03 ± 0.19	0.03 ± 0.11	0.919
Central macular NPA in SCP (mm^2^)	1.52 ± 0.52	1.36 ± 0.46	0.310
Central macular NPA in DCP (mm^2^)	1.80 ± 0.54	1.67 ± 0.53	0.449
Total number of ranibizumab injections administered	5.4 ± 2.4	5.4 ± 2.5	0.994
Occurrence of neovascular changes (%)	6 (17.6%)	0 (0%)	0.030

BRVO = branch retinal vein occlusion; logMAR = logarithm of the minimum angle of resolution; NPA = retinal nonperfusion area; SCP = superficial capillary plexus; DCP = deep capillary plexus; Data are mean ± standard deviation unless otherwise indicated. The baseline measurements of optical coherence tomography angiography were obtained after the initial resolution of macular edema and macular hemorrhage.

At baseline, both CFT and the total NPA were significantly greater in the major BRVO group than in the macular BRVO group (*P* = 0.023 and *P* = 0.001, respectively); however, BCVA showed no significant differences between groups.

At month 12, both the total NPA and the increase in NPA from baseline were significantly larger in the major BRVO group than in the macular BRVO group (50.8 ± 27.9 DA vs. 0.92 ± 2.11 DA and 30.9 ± 22.0 DA vs. 0.02 ± 0.70 DA, respectively, *P* < 0.001 for both). NPA in eyes with macular BRVO showed no significant changes throughout the 24-month observation period. The final BCVA and ranibizumab injection number were not significantly different between the major BRVO and macular BRVO groups. However, the incidence of neovascular changes was significantly higher in the major BRVO group (six eyes, 17.6%) than in the macular BRVO group (0 eyes, 0.0%, *P* = 0.030).

### Comparison of Outcomes Between the Arterial Overcrossing and Venous Overcrossing Groups

To investigate the effects of the vessel position at the affected AV crossing, we compared the clinical outcomes between the arterial overcrossing and venous overcrossing groups (Table 6). To consider the effect of the clinical subtype, we separately investigated outcomes with or without macular BRVO-patients.

**Table 6:** Comparisons of Clinical Characteristics Among Optical Coherence Tomography Angiography-Determined Arteriovenous Crossing Patterns in Eyes with Branch Retinal Vein Occlusion.

	Arterial overcrossing (32 eyes)	Venous overcrossing (23 eyes)	*P* value
Baseline examinations			
LogMAR visual acuity	0.34 ± 0.23	0.24 ± 0.29	0.158
Foveal thickness (μm)	516.3 ± 157.9	498.4 ± 146.8	0.380
Total NPA (disc area)	7.06 ± 12.1	21.5 ± 18.6	0.002
Total NPA (disc area, *only major BRVO*)	14.8 ± 15.6 (12 eyes)	26.9 ± 17.5 (19 eyes)	0.064
Macular NPA in SCP (mm^2^)	1.50 ± 0.42	1.60 ± 0.73	0.656
Macular NPA in DCP (mm^2^)	1.75 ± 0.53	1.75 ± 0.88	0.991
12-months after initial treatment			
LogMAR visual acuity	0.05 ± 0.14	0.02 ± 0.18	0.607
Total NPA (disc area)	16.7 ± 25.6	44.8 ± 29.2	0.002
Total NPA (disc area, *only major BRVO*)	34.7 ± 27.8 (12 eyes)	55.3 ± 22.1 (19 eyes)	0.047
Central macular NPA in SCP (mm^2^)	1.41 ± 0.47	1.71 ± 0.61	0.110
Central macular NPA in DCP (mm^2^)	1.59 ± 0.56	1.94 ± 0.69	0.090
24-months after initial treatment			
LogMAR visual acuity	0.05 ± 0.13	0.01 ± 0.21	0.469
Central macular NPA in SCP (mm^2^)	1.36 ± 0.41	1.59 ± 0.64	0.197
Central macular NPA in DCP (mm^2^)	1.67 ± 0.42	1.81 ± 0.66	0.449
Number of IVR for 24-months	5.6 ± 2.3	4.9 ± 2.4	0.269
Occurrence of neovascular changes	0 (0%)	6 (31.6%)	0.030

Data are mean ± standard deviation unless otherwise indicated; The analyses of total NPA, neovascular complications were examined after exclusion of eyes with macular branch retinal vein occlusion; logMAR = logarithm of the minimum angle of resolution; NPA = retinal nonperfusion area. SCP = superficial capillary plexus; DCP = deep capillary plexus; IVR = intravitreal ranibizumab injections; Total NPAs were separately indicated in all patients, and in patients with major BRVO; The baseline measurements of optical coherence tomography angiography were obtained after the initial resolution of macular edema and macular hemorrhage.

Among the 58 eyes included, 32 (55.2%) showed arterial overcrossing and 23 (39.7%) showed venous overcrossing on OCT angiography (Tables 1 and 6). Three (5.2%) eyes were categorized into the undetermined group, and these were excluded from all analyses comparing clinical characteristics and AV crossing patterns.

**Figure 3:**
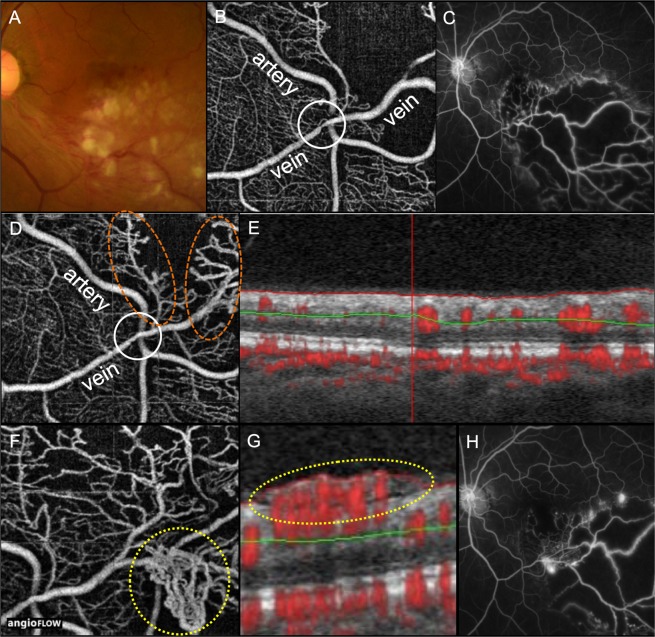
Retinal neovascularization in an eye with major branch retinal vein occlusion (BRVO) and affected venous overcrossing. At baseline (**A**–**C**), a color fundus photograph (**A**), an optical coherence tomography (OCT) angiograph (**B**), and an ultra-wide field fluorescein angiography image (**C**) shows marked retinal whitening, affected venous overcrossing, and a large retinal nonperfusion area (NPA) without any neovascularization, respectively. The initial Snellen visual acuity was 20/67. At month 9 (**D**,**E**), an OCT angiography image shows resolution of narrowing of the affected vein (white circle) and an improvement in the circulation of the venules and capillaries around the affected crossing site (orange circles, **D**). An OCT-B scan of the retinal area showing improved retinal circulation does not show any retinal neovascularization at the vitreoretinal interface (**E**). At month 12 (**F**–**H**), an OCT angiography image clearly shows retinal neovascularization at the periphery of the crossing site (yellow circle, **F**) and the vitreoretinal interface (yellow circle, **G**). An ultra-wide field fluorescein angiography image shows active dye leakage, consistent with retinal neovascularization (**H**). The patient received three ranibizumab injections (no pro re nata injections were necessary) for macular edema. The time between neovascularization and the last ranibizumab injection was approximately 10 months.

At baseline, CFT, BCVA, and the central macular NPA were not significantly different between the two groups. Likewise, the total NPA of eyes with major BRVO were not significantly different between the two groups. At month 12, BCVA, and the central macular NPA showed no significant differences between groups; however, the total NPA was significantly larger in the venous overcrossing group than in the arterial overcrossing group (55.3 ± 22.1 DA vs. 34.7 ± 27.8 DA, *P* = 0.047, Fig. 2). At month 24, BCVA, the central macular NPA, and the ranibizumab injection number did not differ between the crossing patterns, whereas the incidence of neovascular changes was significantly higher in the venous overcrossing group than in the arterial overcrossing group (*P* = 0.030, Fig. 3). None of the six eyes with venous overcrossing and neovascular changes showed PVD.

## Discussion

The current prospective study examined the effects of NPA, the clinical subtype, and the AV crossing pattern on the morphological and functional outcomes, including neovascular changes, of ranibizumab treatment for BRVO-associated ME over an observational period of 24 months.

In the present study, we administered three monthly ranibizumab injections in the first 2 months, followed by PRN (3 + PRN) injections. Similar to findings in previous clinical trials^27–30^, BCVA improved and CFT significantly decreased from baseline during the treatment period (Table 2). The total NPA significantly increased from baseline in eyes with major BRVO, but not in eyes with macular BRVO (Tables 2 and 5, Figs 1 and 2). This suggests that NPAs resulting from BRVO tend to increase in size predominantly in the peripheral retina, not in the central macular area. Under physiological conditions, the retinal artery pressure decreases while the retinal vein pressure increases with an increase in the distance from the optic disc. In eyes with BRVO, the intravascular pressure for the affected vein is considered to increase abnormally in the periphery of the affected crossing site. Therefore, the circulatory status could be expected to deteriorate more in the periphery than in the posterior pole of the eye. Mir *et al*. reported that monthly ranibizumab injections decreased NPA more than PRN injections in eyes with retinal vein occlusion^14^. PRN injections for the treatment of ME may be inadequate for suppressing NPA growth or preventing or reversing capillary closure^31–33^.

Advanced age, an initial poor BCVA, and a greater CFT were associated with a poor final BCVA in our study (Table 3). The central macular NPA at baseline and 12 months was not associated with the final BCVA or the total number of ranibizumab injections administered during the study period (Table 3). In eyes with retinal vein occlusion, the clinical relevance of macular NPA remains controversial. Finkelstein reported that the visual prognosis of eyes with incomplete macular perfusion was better than that of eyes with complete macular perfusion^12^. However, it can be speculated that a macular NPA itself can deteriorate signal phototransduction from the cones to the bipolar and ganglion cells. This deteriorated signal phototransduction may have contributed to the significant negative association between the central macular NPA in the deep capillary plexus (month 24) and the final BCVA in this study (R = 0.364, *P* = 0.019, data not shown).

The total NPA at baseline and 12 months was not associated with the final BCVA or the total number of ranibizumab injections administered during the study period (Table 3). The insignificant association between the total NPA and the 2-year treatment outcome in this study may be supported by the results of other randomized clinical trials^19,20^, where the addition of laser photocoagulation to standard ranibizumab injections did not provide substantial additional benefits with regard to the functional and morphological outcomes, including the treatment burden. To verify the findings of the previous studies and the current study, we compared the treatment outcomes between major BRVO and macular BRVO. The results of these comparisons are presented in Table 5. As expected, patients with major BRVO exhibited larger NPAs than did those with macular BRVO at both baseline and month 12. However, this difference did not appear to affect the functional outcomes or the ranibizumab injection number. The total NPA and intraocular VEGF, which is presumably positively associated with the total NPA, may not be directly related to the central macular function in eyes with BRVO. Negative associations between total NPA and ME have also been reported in diabetic retinopathy^34,35^. Fan *et al*. found that the NPA for the entire retina was not significantly associated with central macular thickness or macular volume^34^. Silva *et al*. also reported that there were no significant associations between NPA and the presence of clinically significant ME or center-involved diabetic ME^35^. Although the pathogenesis differs between diabetic retinopathy and BRVO, these retinal vascular diseases may have a common feature in terms of the association between total NPA and ME.

In addition to the total NPA, other clinical parameters were compared between major BRVO and macular BRVO (Table 5). Interestingly, except for the initial CFT, both CFT and the central macular NPA were comparable between the major BRVO and macular BRVO groups. Hayreh *et al*. reported that major BRVO and macular BRVO are two distinct clinical entities^4,10^. However, Hayreh and Zimmerman also reported that fluorescein dye leakage and microaneurysm formation in the macular area were comparable between major BRVO and macular BRVO groups although these vascular changes at the posterior pole were more severe in the major BRVO group^36^. The functional outcomes of eyes with BRVO might be more strongly related to the central macular perfusion status than to the overall perfusion status. Quite obviously, the area of occlusion in a macular tributary branch vein is more subtle than that in a major arcade vein; therefore, we may have to reconsider the clinical relevance and angiographic features of macular BRVO.

In the present study, six of the 58 eyes (10.3%) showed retinal or disc neovascularization within the 24-month observational period. Previously, the RAVE trial for eyes with severe central retinal vein occlusion reported that VEGF inhibition by ranibizumab injections could delay the occurrence of neovascular complications, although it could not ameliorate the risk of such complications^37^. A regimen involving three initial injections followed by PRN injections, as followed in the present study, may not have been adequate for the complete suppression of neovascular complications in eyes with BRVO. The six eyes that showed neovascular changes in the current study belonged to the major BRVO group, and this is consistent with the findings in previous studies^4,10^. With regard to the incidence of neovascular changes, major BRVO and macular BRVO can be considered distinct entities with regard to the use of anti-VEGF agents for ME treatment. The number of ranibizumab injection required of this study appeared to be smaller compared to those of the previous studies^14,38,39^, a reason of which may be due to strict criteria in the additional ranibizumab injections of this study. However, we considered that it was not undertreatment at least for the treatment ME because the final BCVAs of this study were not inferior to those of previous large cohort study^14,39,40^.

Relative to fluorescein angiography or alternative modalities, OCT angiography is advantageous because of its high depth resolution^40^, which was useful for determining the anatomical positions of the retinal vessels at affected AV crossings in the present study. Similar to the findings in a previous OCT angiography study^25^, we observed that the OCT angiography-determined AV crossing pattern was significantly associated with the total NPA and the central macular NPA (Table 6). Using OCT B-scan images of the affected AV crossing, Muraoka *et al*. reported that occluded veins at the crossing were compressed and obstructed between ILM and the arterial wall in eyes with venous overcrossing, while the venous lumen was generally preserved in eyes with arterial overcrossing^24^. We speculated that these findings could explain the reasons for a larger NPA in eyes with venous overcrossing and a smaller NPA in eyes with arterial overcrossing on OCT angiography. The occluded vein in a venous overcrossing pattern may be too narrow to be clearly detected by previous imaging modalities, and this may be a reason for the disparity in the crossing pattern frequency between previous studies^7–9^ and more recent studies based on OCT or OCT angiography^24,25^.

In our major BRVO group, the final total NPA was significantly larger in eyes with venous overcrossing than in eyes with arterial overcrossing (Table 6). The markedly narrowed veins at the affected AV crossing, as previously demonstrated by OCT B-scans^24^ and OCT angiography^25^, may have contributed to the larger NPAs observed in eyes with venous overcrossing in our study. The crossing pattern were not associated with the final BCVA, and the ranibizumab injection number. However, it was significantly associated with the incidence of neovascular changes. All six eyes with neovascular changes in our study showed venous overcrossing. This suggests that eyes with BRVO and venous overcrossing but no PVD are at a higher risk of neovascularization, regardless of treatment with anti-VEGF injections, if the treatment target is suppression of ME, not neovascularization. To prevent neovascular changes, laser photocoagulation for peripheral NPA may be clinically important as an adjunct to anti-VEGF therapy for the management of ME.

The Branch Vein Occlusion Study previously classified the nonischemic type as that in which the NPA was less than five disc diameters in diameter; the ischemic type was classified as that in which the NPA was more than five disc diameters in diameter, using panoramic images made by five conventional fundus photographs^41^. However, recent clinical settings involving common use of ultra-widefield fluorescein angiography, we have been able to detect peripheral retinal lesions more easily than in previous clinical settings without ultra-widefield fluorescein angiography. If the present study had used the previous criteria, most patients with major BRVO would be classified into the ischemic type. Additionally, in recent BRVO treatments, we have aggressively used anti-VEGF agents that strongly inhibit neovascular complications. In the context of recent clinical settings involving the use of ultra-widefield fluorescein angiography or anti-VEGF agents, the previous criteria^41^ appear to be incompatible with modern clinical practice for BRVO. In this study, we thus quantified the NPA areas and statistically analyzed these measurements without using the previous criteria.

The present study has some limitations. First, the sample size was small and the treatment regimen was the same for all eyes (three injections in the initial 2 months followed by PRN injections). Therefore, we could not compare outcomes by using a consistent monthly injection regimen. In addition, the effect of this anti-VEGF agent on the retinal vasculature remains unknown, and we did not include a control group of eyes that did not receive ranibizumab treatment. Second, unequal magnification is necessary in ultra-widefield imaging, particularly for the peripheral retina, because of the need to accommodate the spherical shape of the fundus. In this study, we measured peripheral NPA without performing any correction because we assumed that the degree of image distortion would be constant among participants. Third, because the status of systemic hypertension was not evaluated in detail, its association with retinal vasculature changes remains unclear. Fourth, to obtain higher-resolution OCT angiograms, we used a viewing angle of 3 × 3-mm^2^ (the smallest viewing angle) and only examined the center of the macular area. However, the macula includes a larger area of the retina than the area sampled in our study. Finally, we did not take intraocular samples or measure VEGF levels in our patient group. Therefore, we were unable to investigate the association between NPA and VEGF levels.

In conclusion, the findings of the current prospective study suggest that advanced age, a poor baseline visual acuity, and a greater CFT are associated with for a worse final BCVA in eyes with BRVO-associated ME treated by ranibizumab (3 + PRN injections). In contrast, the macular NPA or the total NPA may not be associated with the functional outcomes or the treatment burden. These speculations are supported by our finding of no significant differences in clinical outcomes between eyes with major BRVO and those with macular BRVO. Moreover, the total NPA was significantly larger in eyes with venous overcrossing than in eyes with arterial overcrossing. Therefore, the clinical BRVO subtype and the AV crossing pattern may be associated with a risk of neovascular changes, regardless of treatment with ranibizumab injections for ME. An understanding of the association between the NPA size and the AV crossing pattern would contribute to the development of an optimized treatment protocol for individual cases of BRVO. To confirm the current findings and develop a novel treatment regimen for lowering the risk of neovascular complications, further prospective studies should be conducted with a larger cohort and a longer follow-up duration.

## Methods

### Patients

This prospective study was approved by the Institutional Review Board (IRB) of Kyoto University Graduate School of Medicine, Kyoto, Japan, and adhered to the tenets of the Declaration of Helsinki (clinical trial registration numbers: NCT01968616; date of registration: 24/October/2013, last updated: 20/October/2017). Written informed consent was obtained from each participant during the initial visit prior to enrollment in this study.

We included patients who presented at our clinic with acute BRVO involving the macular area, with a symptom duration of less than 4 months before the initial examination, between September 2013 and July 2015. We excluded eyes in which the occluded site was located within the optic disc or on the disc margin. In addition, we excluded eyes with hemicentral retinal vein occlusion, those with multiple retinal vein occlusions, those that received any interventions for ME prior to the study period, those with coexisting ocular diseases (diabetic retinopathy, hypertensive retinopathy, retinal macroaneurysm, glaucoma, or epiretinal membrane), those with keratoconus, those with high myopia (worse than −6 diopters), those with high astigmatism (worse than ±3 diopters), and those with poor-quality OCT angiograms (signal strength index, <50) due to eye movement or media opacities. Eventually, 58 eyes of 58 consecutive patients met these inclusion/exclusion criteria and were included in our study. Because of partial overlap between the recruitment period for the current study and our previously published study, we included three patients from the previous study^25^.

### Examinations and Treatments for ME

At the initial visit, each patient underwent a comprehensive ophthalmic examination, which included measurement of the BCVA using a Landolt chart, 45° digital fundus photography (TRC-50LX; Topcon, Tokyo, Japan; 3,216 × 2,136 pixels), and OCT imaging (Spectralis HRA + OCT, Heidelberg Engineering, Heidelberg, Germany).

All eyes received three monthly ranibizumab injections, and were examined every month for 24 months. We administered additional injections if cystoid spaces and/or serous retinal detachment, extending to the foveal center and the unaffected side became evident on OCT sections. No eyes received any other form of treatment, such as scatter or grid laser photocoagulation, steroid therapy, surgical intervention, or intravitreal injections of anti-VEGF agents other than ranibizumab.

To evaluate the perfusion status of the entire retina, we performed ultra-widefield fluorescein angiography (Optos 200Tx imaging system, Optos PLC, Dunfermline, UK) before and 1 year after the initial ranibizumab injections or when fresh retinal hemorrhage, peripheral white vessels, or neovascular changes appeared during routine ophthalmic examinations.

### OCT Evaluation of the Foveal Thickness

We measured the CFT using macular volume scans obtained by OCT. A whole-retinal thickness map centered on the foveal center was created using the Early Treatment Diabetic Retinopathy Study grid. In cases where we obtained an OCT B-scan with a segmentation error, we manually corrected the delineated line at each B-scan. We obtained retinal thickness values for each grid using the manufacturer’s built-in software (Spectralis Acquisition and Viewing Modules, version 6.0, Heidelberg Engineering, Heidelberg, Germany); CFT was defined as the average value derived from retinal thickness measurements for the central grid.

### OCT Angiography of the Central Macular Area and Affected AV Crossing

We evaluated the central macular perfusion status at every visit by scanning a central 3 × 3-mm^2^ area of the macula using OCT angiography (RTVue XR Avanti, Optovue Inc., Fremont, CA). We also used this tool to classify the relative anatomical vessel positions (arterial or venous overcrossing) at affected AV crossings.

The built-in software in the OCT angiography device automatically recognized the inner limiting membrane (ILM) and inner plexiform layer (IPL). We obtained images of the superficial capillary layer using previously published methods^16,25^. Briefly, the slab containing the superficial capillary plexus was considered to extend from ILM to 15 μm below IPL, while that containing the deep capillary plexus was considered to extend from 15 μm to 70 μm below IPL.

### Measurement of the Central Macular NPA Using OCT Angiography

To avoid segmentation errors in each retinal layer^42^, we measured NPA using OCT angiography when we observed substantial resolution of ME on OCT sections. We defined the central macular NPA as the capillary dropout area within a 3 × 3-mm^2^ section, including the foveal avascular zone. Two fully trained retina specialists (YI-M, YI) blinded to all clinical information about the affected eyes independently reviewed the presence or absence of capillary dropouts in the 3 × 3-mm^2^ section. The central macular NPA was then measured using the manufacturer’s built-in software (AngioVue Clinical Release, v2015.100.0.3, Optovue Inc.). We corrected the NPA measurements for axial length-related magnification using a modified Littmann formula, which applies measurements of the axial length, flatter and steeper meridians, and spherical equivalent (Bennett procedure)^16,25,43^.

The detection of central macular NPAs was partially based on subjective judgments. Therefore, to confirm the validity of central macular NPA measurements in the superficial and deep capillary plexuses, we calculated the ICC for 58 measurements derived for the 58 eyes by three independent examiners.

### Classification of the AV Crossing Pattern Using OCT Angiography

We classified the relative anatomical vessel positions at the affected AV crossings into three groups: arterial overcrossing, venous overcrossing, and undetermined. Arterial overcrossing was defined when the affected artery coursed over the adjacent vein, while venous overcrossing was defined when the affected vein coursed over the adjacent artery. We classified the pattern on the basis of OCT angiograms and the B-scan images of the original OCT scans.

Because classification of the AV crossing pattern was also subjective, we obtained different judgments of the AV crossing pattern in order to avoid misclassification or to minimize classification into the undermined group. Two independent retinal specialists (YI-M and YI) determined the pattern on each image. When the two raters recorded different patterns, a senior retinal specialist (YM) determined the final pattern.

### Measurement of the Total NPA Using Ultra-Widefield Fluorescein Angiography

On ultra-widefield angiography performed 1 min after dye injection, we detected NPA based on subjective judgments for each patient, and measured the NPA size in pixels using a software plugin in the ImageJ program (National Institutes of Health, Bethesda, MD, USA)^25^. For measurement of total NPA, we measured disc area (DA) in pixels for each patient (Supplementary Information), and calculated the mean DA. Using the value of the average DA, total NPA was expressed in units of DA.

To confirm the validity of the total NPA measurements, we calculated the interclass correlation coefficient (ICC) for 58 measurements obtained for the 58 eyes by three independent examiners.

### Determination of the Presence or Absence of Posterior Vitreous Detachment (PVD)

We determined the presence or absence of PVD during the initial examination for each patient. PVD was considered present if we observed a Weiss ring on fundus ophthalmoscopy or ultra-widefield angiography and complete absence of the vitreous cortex on a peripapillary circular OCT scan.

### Statistical Analysis

We statistically analyzed the acquired data using PASW Statistics version 18.0 (SPSS, Chicago, IL). All continuous values are presented as mean ± standard deviation. We obtained BCVA values using a Landolt chart and subsequently converted them to logMAR units for statistical analysis. Comparisons between two groups were performed using unpaired *t*-tests. Differences in each parameter among the different examination time points (baseline and months 12 and 24 after the initial injections) were determined using repeated measures analysis of variance, with a post hoc paired *t*-test used to compare each baseline measurement with each measurement after the initial ranibizumab injections. We used chi-square tests to determine significant differences in the sampling distribution and calculated ICCs to examine the reproducibility and validity of the NPA measurements obtained via fluorescein angiography and OCT angiography. A *P*-value of <0.05 was considered statistically significant. We determined statistical significance after Bonferroni correction for the post hoc test.

YI-M, and YM had full access to all of the data in the study and takes responsibility for the integrity of the data and the accuracy of the data analysis.

## Supplementary information

Supplemental figure
