# Supplementary material for: Branch Retinal Vein Occlusion: Treatment Outcomes According to the Retinal Nonperfusion Area, Clinical Subtype, and Crossing Pattern
Source: Sci Rep. 2019 Apr 25;9:6569. doi: 10.1038/s41598-019-42982-5 (PMC6483995; doi:10.1038/s41598-019-42982-5)
Supplement: Supplementary file 1 — Supplemental figure [file 41598_2019_42982_MOESM1_ESM.pdf]

Branch Retinal Vein Occlusion: Treatment Outcomes According to the Retinal Nonperfusion Area, Clinical Subtype, and Crossing Pattern

Yuko Iida-Miwa, Yuki Muraoka, Yuto Iida,
Sotaro Ooto, Tomoaki Murakami, Kiyoshi Suzuma,
Akitaka Tsujikawa

Supplemental figure 1 S1
How to measure the total NPA on a fluorescein angiography image.

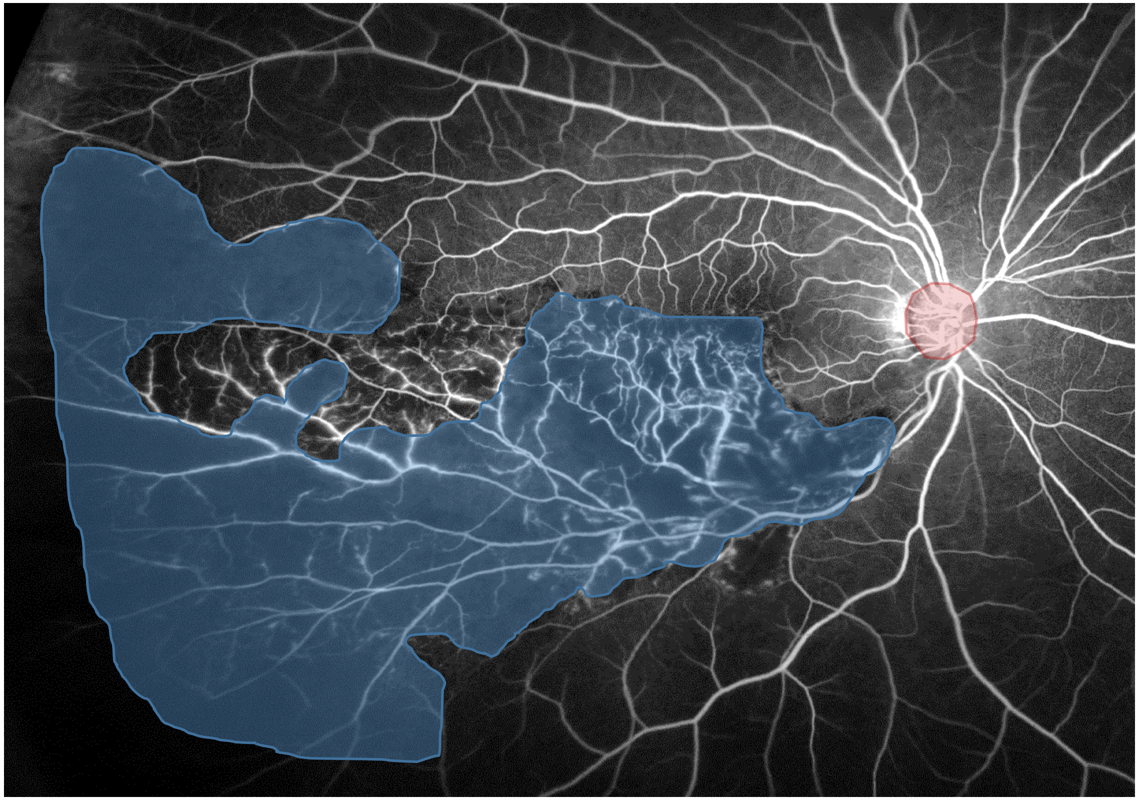

This is a representative case of acute BRVO.
On an image of ultra-widefield angiography obtained 1 minute after dye injection, we detected and measured total NPA (blue area) and disc area (DA) (red area) in pixels using a software plugin in the ImageJ program.
